# The Molecular Balance between Receptor Tyrosine Kinases Tie1 and Tie2 Is Dynamically Controlled by VEGF and TNFα and Regulates Angiopoietin Signalling

**DOI:** 10.1371/journal.pone.0029319

**Published:** 2012-01-03

**Authors:** Harprit Singh, Tania M. Hansen, Nisha Patel, Nicholas P. J. Brindle

**Affiliations:** Department of Cardiovascular Sciences, University of Leicester, Leicester, United Kingdom; University of Illinois at Chicago, United States of America

## Abstract

Angiopoietin-1 (Ang1) signals via the receptor tyrosine kinase Tie2 which exists in complex with the related protein Tie1 at the endothelial cell surface. Tie1 undergoes regulated ectodomain cleavage in response to phorbol esters, vascular endothelial growth factor (VEGF) and tumour necrosis factor-α (TNFα). Recently phorbol esters and VEGF were found also to stimulate ectodomain cleavage of Tie2. Here we investigate for the first time the effects of factors activating ectodomain cleavage on both Tie1 and Tie2 within the same population of cells, and their impact on angiopoietin signalling. We find that phorbol ester and VEGF activated Tie1 cleavage within minutes followed by restoration to control levels by 24 h. However, several hours of PMA and VEGF treatment were needed to elicit a detectable decrease in cellular Tie2, with complete loss seen at 24 h of PMA treatment. TNFα stimulated Tie1 cleavage, and induced a sustained decrease in cellular Tie1 over 24 h whilst increasing cellular Tie2. These differential effects of agonists on Tie1 and Tie2 result in dynamic modulation of the cellular Tie2∶Tie1 ratio. To assess the impact of this on Ang1 signalling cells were stimulated with VEGF and TNFα for differing times and Ang1-induced Tie2 phosphorylation examined. Elevated Tie2∶Tie1, in response to acute VEGF treatment or chronic TNFα, was associated with increased Ang1-activated Tie2 in cells. These data demonstrate cellular levels of Tie1 and Tie2 are differentially regulated by pathophysiologically relevant agonists resulting in dynamic control of the cellular Tie2∶Tie1 balance and modulation of Ang1 signalling. These findings highlight the importance of regulation of signalling at the level of the receptor. Such control may be an important adaptation to allow modulation of cellular signalling responses in systems in which the activating ligand is normally present in excess or where the ligand provides a constitutive maintenance signal.

## Introduction

The angiopoietins are a family of ligands important for blood vessel formation and maintenance [1], [2]. Four angiopoietins have been identified, angiopoietin-1 (Ang1) to -4 and all are secreted glycoproteins of approximately 70 kDa [3]. Ang1 and Ang2 are the best characterized of these ligands and studies with transgenic mice demonstrate both are essential for viability and correct vascular formation [4], [5]. Ang1 acts primarily on endothelial cells to enhance monolayer integrity, suppress apoptosis, stimulate migration and suppress inflammatory gene expression [1], [2]. The actions of Ang1 *in vivo* reflect these cellular activities and the ligand is a potent inhibitor of vascular leakage and inflammation as well as promoting microvessel survival [1], [2]. In addition to roles in angiogenesis, therefore, Ang1 has key functions in vascular maintenance. The ligand is expressed at relatively constant rates by perivascular cells and it acts on underlying endothelium where it maintains its receptor, Tie2, in a state of activation [6]. This results in a sustained pro-stabilizing effect on the vasculature, suppressing vessel leakage and inflammatory activation [1], [2]. Ang2 is a context dependent antagonist of Ang1, suppressing binding of Ang1 to its primary receptor, Tie2, blocking Ang1 signalling and functional effects [7]. Consistent with this antagonistic activity the phenotype of transgenic mice overexpressing Ang2 is similar to Ang1 knockout mice [7].

The primary receptor for angiopoietins, Tie2, is a receptor tyrosine kinase expressed predominantly on endothelial cells [8]. Binding of Ang1 to Tie2 enhances tyrosine kinase and tyrosine phosphorylation of the receptor resulting in recruitment of signalling molecules, including the p85 subunit of phosphatidylinositol-3-kinase PI-3K, A20 binding inhibitor of NFkB-2, Dok-R and Grb2 [1], [2], [9]. Downstream signalling pathways activated by Tie2 include the PI-3K/Akt pathway which has been implicated in the pro-survival, migratory and anti-inflammatory activities of Ang1 and Erk1/2 pathways2 [1], [2], [9]. Tie2 interacts at the cell surface with the related receptor tyrosine kinase Tie1 [10]. The extracellular domains of Tie1 and Tie2 are similar in overall structure, comprising of two immunoglobulin-like domains followed by three EGF-homology sequences, another immunoglobulin-like domain and three fibronectin III domains before the transmembrane sequence [11], [12], [13], [14], [15], [16]. However, the amino acid identity between the two receptor ectodomains is only around 30% [11], [12], [13], [14], [15], [16] and Tie1 is unable to bind angiopoietins [8]. Indeed, Tie1 inhibits Ang1 signalling through Tie2 [17], [18].

Recently it has been reported that the extracellular ligand-binding domain of Tie2 undergoes proteolytic cleavage [19], [20], [21]. Tie2 cleavage has been identified by the observed decrease in full-length receptor and the discovery of soluble Tie2 fragment of approximately 75–85 kDa in size in conditioned media from endothelial cells [19]. Cleavage was found to occur over a period of hours constitutively and was activated by phorbol esters and vascular endothelial growth factor (VEGF) treatment [19], [20]. Importantly, Tie2 ectodomain cleavage has been shown to occur also *in vivo* and a soluble extracellular Tie2 fragment has been observed in blood from healthy volunteers [19]. Cleavage of Tie2 inhibits the ability of Ang1 to bind and activate signalling. In addition, the released soluble Tie2 fragment is to bind and sequester Ang1 and prevent interaction and signalling by any remaining full-length receptor in the cell membrane. Soluble Tie2 has been shown to bind both Ang1 and Ang2 and decrease the observed phosphorylation of Tie2 induced by these ligands in non-endothelial cells expressing Tie2 receptors [20]. Therefore, activation of Tie2 cleavage may be an important mechanism by which Ang1 signalling is regulated by stimuli such as VEGF.

Like Tie2, Tie1 also undergoes regulated ectodomain cleavage in which the extracellular domain of Tie1 is released by metalloprotease activity [22], [23], [24]. This occurs under basal conditions and is activated by a range of stimuli such as phorbol esters, VEGF, tumour necrosis factor-α and changes in shear stress [22], [23], [24], [25]. Released Tie1 ectodomain is present in blood [24]. Activated Tie1 cleavage occurs rapidly over a period of minutes [17]. Importantly, as Tie1 ectodomain is unable to bind Ang1 the released extracellular part of this receptor does not suppress Ang1 action. Indeed, in contrast to Tie2 cleavage, loss of Tie1 or Tie1 ectodomain actually enhances Ang1 signalling [17], [18]. This occurs because in the Tie1∶Tie2 complexes that exist at the cell surface the extracellular domain of Tie1 restricts access of Ang1 to Tie2 and loss of Tie1 ectodomain allows increased Ang1 binding to Tie2 [17].

Taken together the data on Tie1 and Tie2 cleavage raise questions about the effects of stimuli such as VEGF on Ang1 action. Induction of Tie1 cleavage by VEGF would be expected to enhance Ang1 activation of Tie2, and this has indeed been demonstrated [17]. However as this stimulus activates Tie2 cleavage it would be expected also to suppress Tie2 signalling [20]. These two opposing actions could be reconciled if they occurred with different kinetics, with an acute increase in Tie2∶Tie1 ratio due to Tie1 cleavage leading to enhancement of Ang1 signalling, followed by loss of Tie2 causing a decrease in Tie2∶Tie1 and suppressing Ang1 signalling if the stimulus is maintained. The published data does suggest Tie1 cleavage occurs more rapidly than Tie2, however the kinetics of cleavage of both receptors has not been examined in the same population of cells. Here for the first time we directly examine activated Tie1 and Tie2 cleavage in human endothelial cells and the impact of this on cellular Ang1 signalling. Our data show that cleavage of each receptor is differentially controlled and that this results in dynamic regulation of the cellular Tie2∶Tie1 by pathophysiologically relevant factors resulting in modulation of Ang1 signalling.

## Results

### Kinetics of Tie1 and Tie2 cleavage

**Figure 1:**
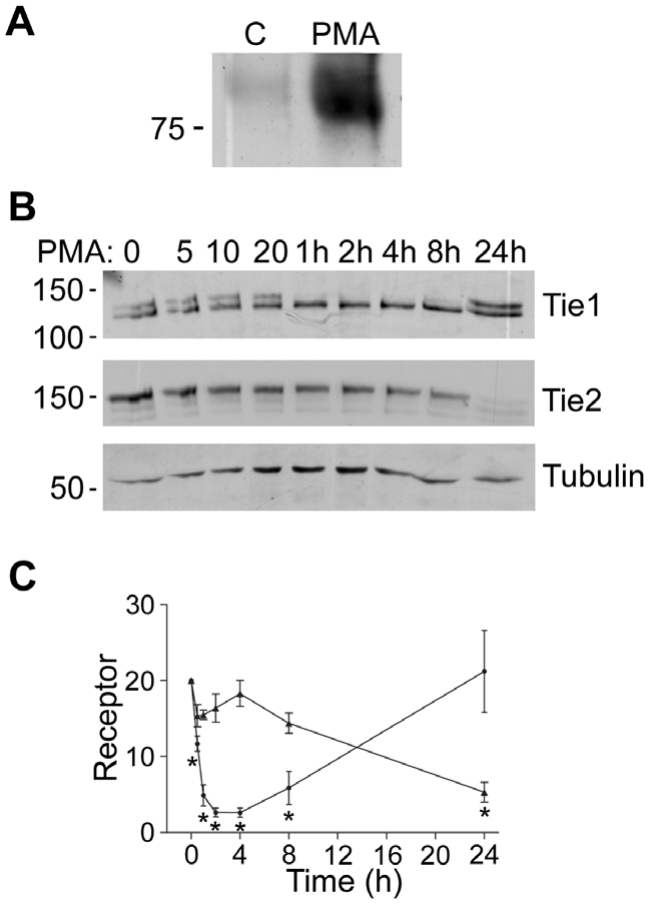
Kinetics of Tie1 and Tie2 Cleavage. A Tie2 extracellular domain is released from endothelial cells. HUVEC were untreated or stimulated with 10 ng/ml PMA for 24 h, as indicated, before collection of culture medium, removal of cellular material by centrifugation and immunoprecipitation of Tie2 extracellular domain. Immunoprecipitates were resolved by SDS/PAGE and released Tie2 extracellular domain detected by immunoblotting. The position of a 75 kDa molecular mass marker is indicated. B Time course of the effects of PMA on cellular full-length Tie1 and Tie2. Endothelial cells were incubated with 10 ng/ml PMA for the times indicated in minutes or hours (h) before cell lysis and detection of cellular Tie1 by immunoblotting. To determine levels of full-length Tie2 in the same cell population, blots were stripped and re-probed for Tie2. Blots were also re-probed for tubulin. The relative mobility of mass markers is shown in kDa. C Full-length Tie1 (black circle) and Tie2 (black triangle) in endothelial cells treated with 10 ng/ml PMA for different times were determined by immunoblotting in three independent experiments and quantitated by densitometric scanning. Data are means and SEM and are presented as arbitrary units normalized to the levels in untreated cells within each experiment. The asterisk indicates a statistically significant effect of PMA (P<0.05, Students ‘t’ test).

Cleavage of both Tie1 and Tie2 has been reported to be strongly activated by PMA in endothelial cells [19], [20], [23]. Cleavage of both receptors in the same population of cells therefore was initially examined in response to PMA. To confirm that Tie2 cleavage occurred under our experimental conditions HUVEC were challenged with PMA for 24 hours and conditioned medium for the cells probed for the presence of Tie2 ectodomain by immunoblotting. As shown in figure 1A, Tie2 ectodomain was barely detectable in medium from control cells but was clearly evident in the medium taken from cells treated with PMA, consistent with previous data [19], [20]. Next we examined the effects of PMA on cellular levels of Tie1 and Tie2 in the same populations of HUVEC. To do this HUVEC were incubated in the absence of PMA or with PMA for increasing time, up to 24 hours, and the levels of each receptor examined in the cells by immunoblotting. As described previously [23], Tie1 is seen as a doublet of approximately 145 kDa which corresponds to surface expressed fully glycosylated receptor and a smaller band of partially glycosylated receptor destined for the cell surface [22]. PMA activates cleavage of full-length glycosylated Tie1, seen as loss of the upper band of the doublet; this is clearly evident at 1 h of PMA treatment and continues over the following 7 h (Fig. 1B, C). By 24 h of PMA treatment cellular Tie1 levels have recovered and the receptor is found as full-length, uncleaved Tie1 in the cells. These data are consistent with previous reports showing similar levels of Tie1 in HUVEC treated for 24 h with PMA as in untreated cells [23]. In contrast to this, there was little loss of Tie2 in HUVEC over the initial minutes of PMA treatment but loss of full-length Tie2 could be clearly seen by 24 hours (Figure 1B, C). These data show that there is an initial acute drop in levels of full-length Tie1 in response to PMA whilst Tie2 remains relatively unchanged, whereas by 24 h Tie1 levels are restored and Tie2 is clearly diminished (Figure 1C).

**Figure 2:**
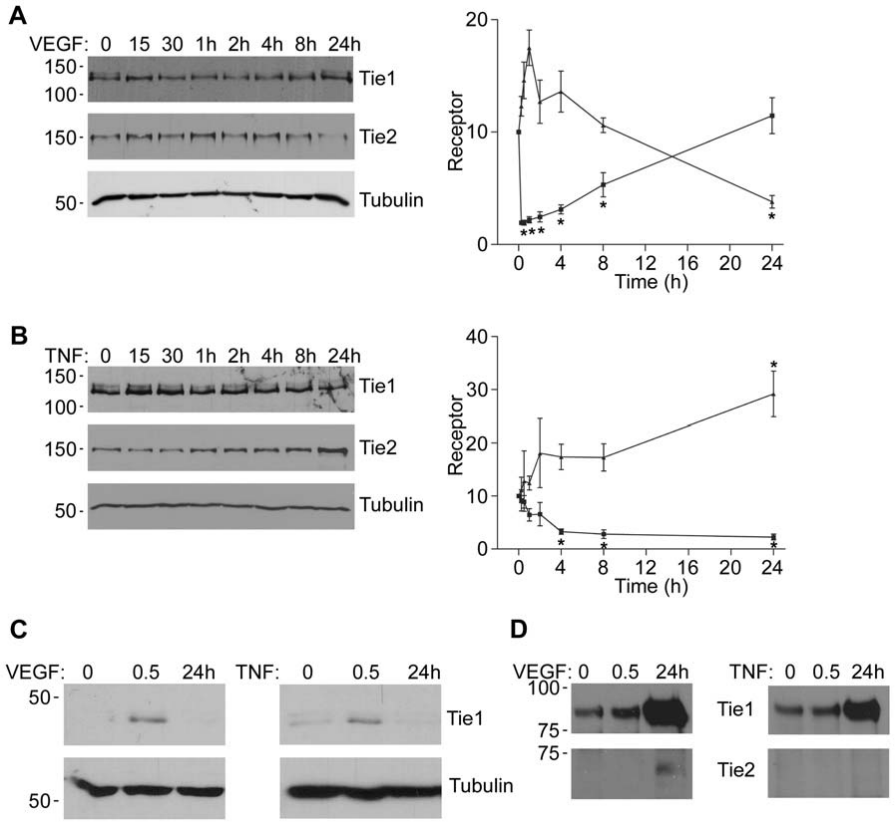
Effects of VEGF and TNFá on Tie1 and Tie2 Cleavage. HUVEC were incubated with VEGF A or TNFα B for times up to 24 h as indicated before cell lysis and detection of cellular full-length Tie1 by immunoblotting. Blots were stripped and re-probed for full-length Tie2 and tubulin, as indicated. Full-length Tie1 and Tie2 in endothelial cells treated with VEGF or TNFα at the different times points was determined by immunoblotting in at least three independent experiments and quantitated by densitometric scanning. Data are means and SEM and presented as arbitrary units normalized to the levels in untreated cells within each experiment. Asterisks indicate significant effect compared with untreated cells (p<0.05, Student's ‘t’test). C VEGF and TNFα stimulate formation of the intracellular Tie1 fragment. HUVEC were incubated with VEGF or TNFα for 30 min or 24 h as indicated before cell lysis and detection of cellular Tie1 endodomain by immunoblotting with an antibody recognizing the Tie1 C-terminus. Blots were stripped and re-probed for tubulin, as indicated. D VEGF and TNFα stimulate release of Tie1 and Tie2 ectodomain from endothelial cells. HUVEC were untreated or stimulated with VEGF or TNFα for 30 min or 24 h, as indicated, before collection of culture medium, removal of cellular material by centrifugation and immunoprecipitation of Tie1 and Tie2 extracellular domains. Immunoprecipitates were resolved by SDS/PAGE and released extracellular domains detected by immunoblotting. The relative mobility of mass markers is shown in kDa.

VEGF is a key physiological activator of both Tie1 and Tie2 cleavage with similar, though less marked, effects as PMA [20], [22], [26]. Therefore we tested whether, like PMA, the effects of VEGF on cellular Tie1 and Tie2 occurred at early and late time points respectively. To do this HUVEC were stimulated with a similar concentration of VEGF as previously described [22], [26] for various times up to 24 hours and cellular Tie1 and Tie2 determined by immunoblotting (Fig. 2A). At 30 min loss of cellular full-length Tie1 protein is seen and levels are restored at 24 h (Fig. 2A). In contrast, and similar to PMA, levels of cellular Tie2 after 30 min of VEGF stimulation are similar to control levels, whereas by 24 h of VEGF stimulation cellular Tie2 is significantly decreased (Fig. 2A). VEGF therefore increases the cellular ratio of full-length Tie2∶Tie1 acutely but decreases the ratio chronically.

TNFα is also an important activator of Tie1 cleavage in endothelial cells [22]. However its effects on Tie2 have not been described. We therefore examined the effects of TNFα on Tie1 and Tie2 at various times up to 24 h. As shown in figure 2B, TNFα caused a progressive loss of full-length Tie1 from 30 min and becoming statistically significant at 4 h. In contrast to PMA and VEGF, TNFα caused a substantial decrease in cellular Tie1 at 24 h (Fig. 2B). Interestingly, in addition to a decrease in the level of full-length surface expressed Tie1, the level of the lower molecular mass partially glycosylated form of Tie1 was also found to be decreased at 24 h. This form of Tie1 is an intracellular precursor of full-length Tie1 [23]. The decrease in Tie1 precursor induced by 24 h of TNFα suggests that at this extended time point the ligand may decrease Tie1 expression. In the same population of cells TNFα had no detectable effect on cellular Tie2 levels at 30 min. In addition, this cytokine caused a small, statistically significant, increase in Tie2 at 24 h (Fig. 2B). This stimulatory effect of TNFα is consistent with a previous report demonstrating upregulation of Tie2 by TNFα [27]. Overall and in contrast to PMA and VEGF, therefore, this inflammatory cytokine causes an increase in cellular Tie2∶Tie1 ratio chronically.

The data presented in figure 2B suggest that whilst loss of full-length Tie1 at early time points likely reflects the ability of this ligand to stimulate cleavage, as previously reported [22], loss at 24 h may not be due to cleavage but decreased expression. Ectodomain cleavage of Tie1 results in formation of a 45 kDa cell-associated fragment of the receptor comprising of transmembrane and intracellular domains [22]–[24]. Therefore to test whether TNFα induces cleavage at 30 min and 24 h cells were treated with the ligand for the relevant times, followed by cell lysis, blotting and probing of lysates with an antibody that detects the Tie1 intracellular domain (Fig. 2C). TNFα clearly induces formation of the 45 kDa intracellular domain fragment at 30 min but not after 24 h treatment indicating the acute decrease in full-length Tie1 reflects cleavage whereas 24 h effects are not due to cleavage. For completeness similar experiments were performed with VEGF (Fig. 2C) and revealed an increase in Tie1 endodomain at 30 min but not 24, as expected for the acute stimulation of Tie1 cleavage induced by VEGF.

We also examined levels of released Tie1 and Tie2 ectodomain at early (30 min) and late (24 h) time points in cells treated with VEGF and TNFα. As shown in figure 2D, VEGF stimulated an increase in soluble Tie1 ectodomain at 30 min of stimulation. Levels of Tie1 ectodomain in the medium was further elevated at 24 h of VEGF treatment, consistent with Tie1 cleavage occurring over the first few hours of VEGF treatment (Fig. 2A). Levels of soluble Tie2 ectodomain in the medium were not detectable after 30 min of VEGF treatment but was clearly seen at 24 h (Fig. 2D), as expected with the increased time required for VEGF to induce significant Tie2 cleavage (Fig. 2A, B). Treatment of endothelial cells with TNFα caused detectable soluble Tie1 ectodomain in the medium after 30 min and substantial levels at 24 h (Fig. 2D). This effect of TNFα on release of soluble ectodomain suggests the ligand activated Tie1 cleavage at 30 min and that this is sustained over the first few hours of TNFα treatment, as shown in figure 2B. In the case of Tie2 ectodomain, and consistent with the lack of effect of TNFα on Tie2 cleavage, released Tie2 ectodomain was not detected in medium of cells treated acutely (30 min) or chronically (24 h) with the ligand (Fig. 2D).

Taking the findings on full-length cellular Tie1 and release of ectodomain together these data indicate that VEGF activates Tie1 cleavage acutely but not chronically, whereas TNFα causes Tie1 cleavage acutely and further loss of Tie1 chronically, via effects nt involving cleavage. Furthermore, chronic, but not acute, VEGF treatment stimulates loss of Tie2 whereas chronic TNFα treatment elevates cellular Tie2. The net result of these ligands therefore is for VEGF to decrease full-length Tie1∶Tie2 ratio acutely and increase the ratio chronically whereas TNFα causes a clear decrease in Tie1∶Tie2 ratio with increasing time of treatment.

### Tie2 Endodomain

**Figure 3:**
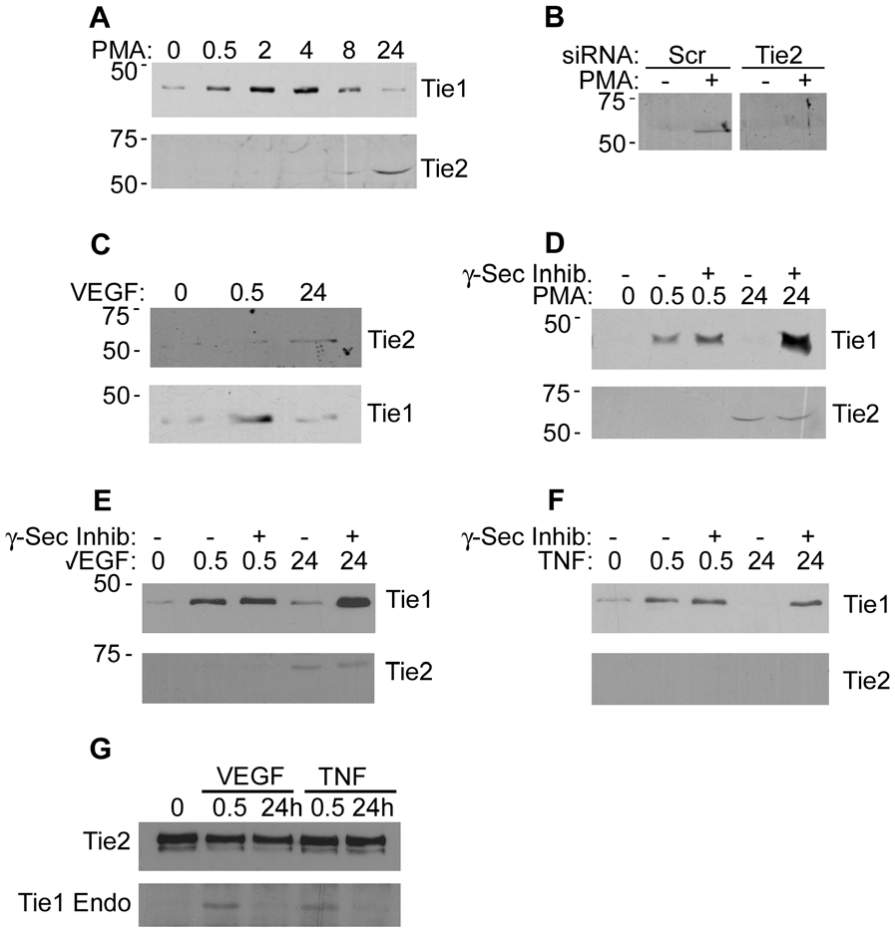
Detection of Tie2 Cell-Associated Endodomain. A Cells were incubated with PMA for the times indicated in hours before cell lysis and detection of cellular Tie1 endodomain by immunoblotting with an antibody recognizing Tie1 carboxy-terminus, as indicated. Blots were stripped and re-probed with an antibody to Tie2 intracellular domain, as indicated. The positions of molecular mass markers in kDa are shown on the left of the blots. B To confirm the 55 kDa Tie2 immunoreactive fragment is derived from Tie2 endothelial cells were transfected with control siRNA (Scr) or siRNA recognizing Tie2, as indicated. 24 h post-transfection cells were incubated with or without PMA for 24 h before lysis and detection of Tie2 endodomain by immunoblotting. C VEGF stimulates Tie2 endodomain accumulation at 24 h. Endothelial cells were incubated with VEGF for 0.5 or 24 h before cell lysis and detection of cellular Tie2 intracellular domain. Membranes were stripped and reprobed for Tie1 intracellular domain. D Effects of γ-secretase inhibition on Tie1 and Tie2 endodomains formed in response to PMA. HUVEC were incubated with or without the γ -secretase inhibitor in the absence and presence of PMA for 0.5 or 24 h as indicated. Tie1 endodomain was detected in whole cell lysates by immunoblotting with an antibody recognizing Tie1 carboxy-terminus and blots were stripped and re-probed with an antibody to Tie2 intracellular domain as indicated. Effects of γ-secretase inhibition on Tie1 and Tie2 endodomains formed in response to VEGF (E) and TNFα (F). HUVEC were incubated with or without the γ -secretase inhibitor in the absence and presence of VEGF or TNFα for 0.5 or 24 h as indicated. Tie1 endodomain was detected in whole cell lysates by immunoblotting with an antibody recognizing Tie1 carboxy-terminus and blots were stripped and re-probed with an antibody to Tie2 intracellular domain as indicated. G Tie1 endodomain interacts with full-length Tie2. HUVEC were incubated with VEGF or TNFα for 0.5 or 24 h as indicated. Cells were lysed and Tie2 immunoprecipitated. Tie1 endodomain co-immunoprecipitating with Tie2 was detected by immunoblotting with an antibody recognizing Tie1 carboxy-terminus and blots were stripped and re-probed with an antibody to Tie2. The relative mobility of mass markers is shown in kDa.

The cell-associated fragment of Tie1 remaining after ectodomain cleavage encompasses transmembrane and intracellular domains and is clearly observed as an approximately 45 kDa protein that accumulates in endothelial cells following activation with PMA, VEGF and other stimuli [22], [24]. We were interested to determine whether the Tie2 fragment generated after Tie2 ectodomain cleavage could be detected in endothelial cells. HUVEC were therefore treated for 30 min or 24 h with PMA before analysis of cell lysates for the Tie1 and Tie2 proteolytic fragment by immunoblotting with antibodies recognizing the carboxy-terminus of the intracellular domains (Fig. 3A). As expected from previous studies the 45 kDa Tie1 fragment is observed within minutes of treatment of cells with PMA (Fig. 3A). Consistent with the requirement for more chronic stimulation to induce appreciable cellular Tie2 cleavage, a 55 kDa Tie2 immunoreactive protein appears in cell lysates following 24 h of PMA treatment (Fig. 3A). In order to confirm that the 55 kDa protein was derived from Tie2 we used siRNA to suppress Tie2 expression and examined the appearance of the fragment in response to 24 h PMA treatment. HUVEC were transfected with control siRNA or siRNA targeting Tie2 and cultured for 24 h then treated with PMA for an additional 24 h before cell lysis and detection of Tie2 by immunoblotting (Fig. 3B). Cells transfected with siRNA targeting Tie2 expressed undetectable levels of the 55 kDa protein following 24 h PMA treatment whereas this protein was still apparent in similarly treated cells transfected with control siRNA (Fig. 3B). These data, together with the correlation in kinetics of appearance of the 55 kDa with the loss of full-length Tie2, plus the immunoreactivity of the 55 kDa protein with an antibody recognizing the Tie2 intracellular domain, are consistent with the 55 kDa protein being the cell-associated Tie2 cleavage product.

As a physiological activator, VEGF induces a more modest Tie2 cleavage than PMA and it was important to test whether this physiologically relevant stimulus is sufficient to induce appearance of the Tie2 endodomain. Therefore HUVEC were stimulated for 30 min and 24 h with VEGF before analysis for the presence of Tie2 endodomain by immunoblotting (Fig. 3C). As with PMA, the 55 kDa Tie2 fragment is not observed at 30 min whereas 24 h treatment with VEGF results in the appearance of the 55 kDa Tie2 fragment (Fig. 3C). Probing blots for Tie1 endodomain revealed an increase in Tie1 cellular Tie1 endodomain after 30 min of VEGF treatment and loss by 24 h, consistent with acute stimulation of cleavage by VEGF causing increased endodomain levels, followed by degradation of the newly formed endodomain [17].

It has previously been shown that once formed by ectodomain proteolysis Tie1 endodomain is degraded by a two step process involving release of the intracellular domain from the membrane tethered transmembrane sequence, by γ-secretase activity, followed by proteosomal degradation of the 42 kDa intracellular kinase domain [17]. Consequently, inhibition of γ-secretase results in the accumulation the 45 kDa Tie1 endodomain [17]. A similar two-step degradation occurs for a number of other receptor tyrosine kinases undergoing regulated ectodomain cleavage, including ErbB4 and colony-stimulating factor-1 receptor [28], [29]. It was therefore of interest to determine whether the Tie2 endodomain is also a substrate for γ-secretase-mediated degradation. To test this HUVEC were incubated in the absence and presence of the γ-secretase inhibitor DAPT (N-[N-(3,5-Diflurophenacetyl-L-alanyl])-S-phenylglycine *t*-Butyl-Ester), treated with or without PMA and Tie1 and Tie2 detected in cell lysates by immunoblotting. As shown in figure 3D, inclusion of PMA activated formation of the 45 kDa endodomain fragment of Tie1 at 30 min and the γ-secretase inhibitor increased the level of this endodomain. Interestingly, there is also a clear accumulation of the Tie1 endodomain fragment in cells incubated with γ-secretase inhibitor for 24 h, presumably reflecting inhibition of degradation of the endodomain produced at basal rates and induced by PMA acutely earlier in this 24 h period. In contrast, although PMA stimulated formation of the 55 kDa Tie2 fragment by 24 h this was not affected by the presence of γ-secretase inhibitor (Fig. 3D). This suggests that Tie2 endodomain is not a substrate for γ-secretase activity. In further work we examined the effects of γ-secretase on endodomains induced by VEGF and TNFα (Fig. 3E, F). VEGF stimulates formation of Tie1 endodomain at 30 min and this was enhanced by γ-secretase inhibitor. There was no increase in Tie1 endodomain in response to 24 h of VEGF treatment, although, as in the case of the PMA experiments, the presence of γ-secretase inhibitor for 24 h did increase the level of endodomain seen in the cells, reflecting accumulated endodomain from acute VEGF treatment. VEGF stimulates formation of Tie2 endodomain at 24 h but not 30 min, and γ-secretase inhibitor did not affect this (Fig. 3E). TNFα stimulated Tie1 endodomain formation at 30 min and, as with VEGF and PMA, in the presence of γ-secretase inhibitor this was further elevated (Fig. 3F). After 24 h TNFα treatment there was no elevation of Tie1 endodomain but, again, increased endodomain after 24 h in the presence of γ-secretase inhibitor (Fig. 3F). Consistent with the lack of effect of TNFα on Tie2 cleavage, TNFα did not stimulate formation of Tie2 endodomain either in the absence or presence γ-secretase inhibitor (Fig. 3F). Together these data show that whereas Tie1 endodomain is subject to degradation by γ-secretase, this does not appear to be the case for Tie2 endodomain.

Previously we reported the presence of complexes comprising full-length Tie2 physically associated with Tie1 endodomain in endothelial cells treated with VEGF [26]. We were therefore interested to see if such complexes were present in cells treated chronically with VEGF as well as acutely and chronically with TNFα. To do this endothelial cells were treated with VEGF or TNFα for 30 min or 24 h before lysis and immunoprecipitation of Tie2. Immunoprecipitates were then probed for the presence of associated Tie1 endodomain. As shown in figure 3G, Tie2 was found interacting with Tie1 endodomain after 30 min treatment of cells with VEGF as well as TNFα. However, such complexes were not observed in cells treated for 24 h with either VEGF or TNFα, reflecting the lack of Tie1 endodomain in cells treated with either ligand for 24 h (Fig. 2C, 3E, F).

### Effects of Tie1 on Ang1 signalling

**Figure 4:**
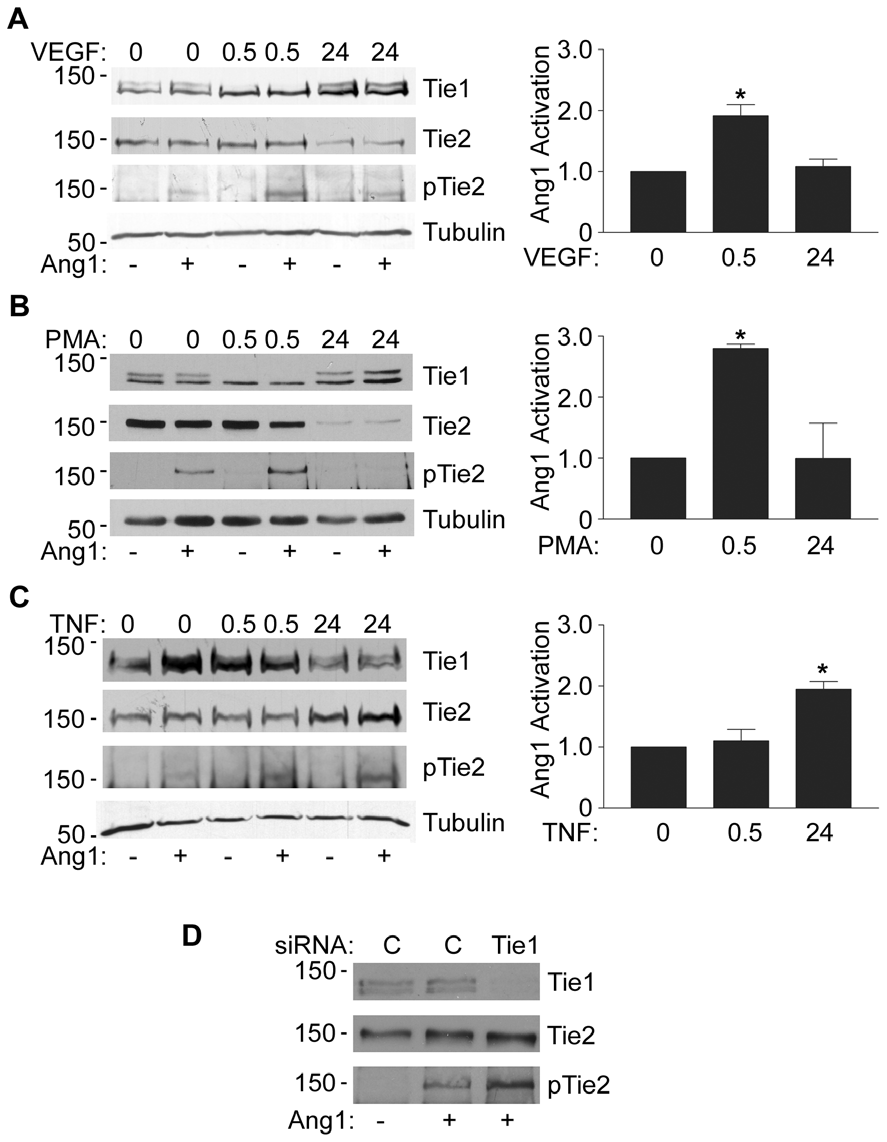
Effects of VEGF, PMA and TNFá on Cellular Tie2 Activation. HUVEC were treated with VEGF A, PMA B or TNFα C for 0, 0.5 or 24 h to induce Tie1 and Tie2 ectodomain cleavage followed by 200 ng/ml Ang1 for 30 min. Cells were lysed and phosphorylated Tie2 detected by immunoblotting with anti-phospho-Tie2 (pTie2), blots were stripped and re-probed for Tie1, Tie2 and tubulin. Phosphorylated Tie2 in cells treated with VEGF, PMA or TNFα for 0, 0.5 or 24 h and activated with Ang1 was detected by immunoblotting in at least three independent experiments and quantitated by densitometric scanning. Data are means and SEM of phospho-Tie2 normalized to that in Ang1-activated control cells within each experiment. Asterisks indicates significant increase in Ang1-induced Tie2 phosphorylation (p<0.05, Student's ‘t’test). D Loss of Tie1 enhances Ang1-activation of Tie2. HUVEC were transfected with contraol siRNA (C) or siRNA targeting Tie1 (Tie1) as indicated. 24 h post-transfection cells were activated with Ang1 for 30 min before lysis and detection of phosphorylated Tie2 (pTie2), blots were stripped and re-probed for Tie1 and Tie2. The relative mobility of mass markers is shown in kDa.

We and others have shown that loss of full-length Tie1 or cleavage of Tie1 ectodomain enhances Ang1 signalling through Tie2 [17], [18]. In contrast, loss of Tie2 ectodomain would be expected to suppress Ang1 signalling through Tie2 by two routes, loss of the ligand-binding domain from the receptor plus sequestration of Ang1 by released ectodomain. Indeed, Findley et al have recently reported suppression of Ang1-activation of Tie2 by Tie2 ectodomain released by PMA-induced Tie2 ectodomain cleavage [20]. We hypothesized, therefore, that stimuli that activate Tie1 and Tie2 cleavage with differing kinetics, such as VEGF, would regulate Ang1-induced Tie2 signalling. To test this HUVEC were subjected to acute and chronic stimulation with VEGF before addition of Ang1 for 30 min. Cells were then lysed and levels of Tie2 phosphorylation in cells determined by immunoblotting lysates with anti-phospho-Tie2. As shown in figure 4A, VEGF treatment for short periods (30 min) increased Ang1-induced Tie2 phosphorylation in endothelial cells, consistent with previous reports [17]. However, after 24 h of VEGF treatment this enhanced cellular Tie2 signal was decreased as Tie1 levels were restored and Tie2 cleaved (Fig. 4A). Similar experiments were also performed to examine the effects of PMA-induced Tie1 cleavage on Ang1-activation of Tie2 (Fig. 4B). As with VEGF, treatment of cells with PMA for 30 min caused cleavage of full-length Tie1 and enhanced Ang1-activated Tie2 phosphorylation, whereas by 24 h of PMA treatment full-length Tie1 was restored, Tie2 decreased and the elevated Ang1-induced Tie2 phosphorylation decreased (Fig. 4B).

As shown in figure 3, TNFα induces a chronic depression of cellular full-length Tie1. This result in an increase in the molecular ratio of Tie2∶Tie1 in cells chronically. These effects would be predicted to increase Ang1-activated Tie2 at 24 h. To test this, endothelial cells were activated for 30 min or 24 h with TNFα before stimulating for 30 min with Ang1 and assessment of Tie2 phosphorylation by immunoblotting. As predicted TNFα caused a significant increase in the level of Ang1-induced Tie2 phosphorylation in cells at 24 h (Fig. 4C). There was little effect of 30 min TNFα-treatment on Ang1-activated Tie2 phosphorylation, consistent with the finding that although Tie1 cleavage has been initiated (Fig. 2D) at 30 min there is still appreciable full-length Tie1 present in the cells (Fig. 2B, 4C). We also confirmed that loss of Tie1 enhances Ang1-activation of Tie2 by using an siRNA approach. In these experiments endothelial cells were transfected with control siRNA or siRNA targeting Tie1. After 24 h cells were stimulated with Ang1 and levels of phosphorylated Tie2 examined by immunoblotting as above. Cells transfected with siRNA targeting Tie1 showed a loss of the receptor and an enhancement of Ang1-activated Tie2 phosphorylation (Fig. 4D).

### Phosphorylation of Tie2 endodomain

**Figure 5:**
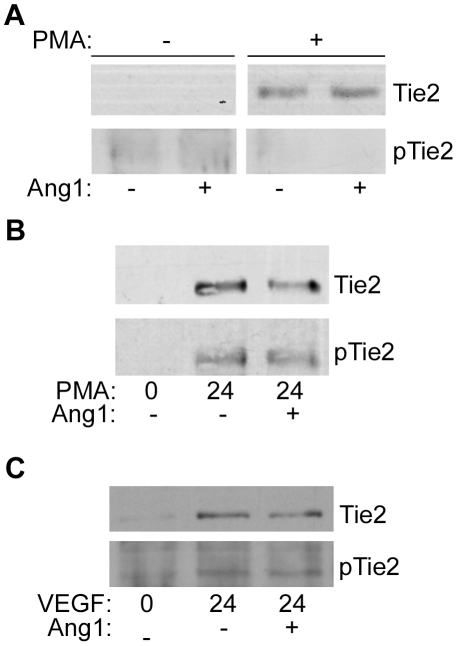
Phosphorylation of Tie2 Endodomain. A Endothelial cells were incubated for 24 h in the absence of presence of PMA, as indicated, before stimulation with Ang1 for 30 min. Cell lysates were resolved by SDS/PAGE, transferred to nitrocellulose and probed for phospho-Tie2 (pTie2) and Tie2 as indicated. B Following stimulation with PMA for 24 h endothelial cells were incubated with or without Ang1 for 30 min, as indicated. Cells were lysed and Tie2 endodomain recovered from lysates by immunoprecipitation with an antibody recognizing Tie2 intracellular domain and phosphorylation of Tie2 endodomain determined by anti-phosphotyrosine immunoblotting. Blots were stripped and re-probed for Tie2 endodomain. C HUVEC were treated with VEGF for 24 h, with or without Ang1 for 30 min, as indicated. Cells were lysed and Tie2 endodomain recovered from lysates by immunoprecipitation with an antibody recognizing Tie2 intracellular domain and phosphorylation of Tie2 endodomain determined by anti-phosphotyrosine immunoblotting. Blots were stripped and re-probed for Tie2 endodomain. The relative mobility of mass markers is shown in kDa.

In addition to signalling from full-length Tie2 it is possible that the Tie2 intracellular domain fragment detected in cells following ectodomain cleavage could have signalling activity. This could occur in the absence of Ang1 as a result of loss of the Tie2 regulatory ectodomain. Indeed, some other intracellular kinase domains released following ectodomain cleavage of full-length receptor tyrosine kinases, such as ErbB4 and TrkA, appear to be active as judged by their phosphorylation status [30], [31]. To test this, endothelial cells were treated with phorbol ester for 24 h before stimulation with Ang1 for 30 min. Lysates were resolved by SDS/PAGE and probed for activated phosphorylated Tie2 endodomain and total Tie2 endodomain by immunoblotting (Fig. 5A). As before, Tie2 endodomain was detectable after 24 h of PMA treatment. However, phosphorylation of the endodomain was not detectable in untreated cells or following Ang1-activation. It is possible that Tie2 endodomain is phosphorylated at a low level that is not detectable by anti-phospho-Tie2 immunoblotting of whole cell lysates. In order to increase the possibility of detecting phosphorylation of Tie2 endodomain, therefore, cells were treated with PMA and Ang1 as before and following lysis Tie2 intracellular domain recovered from cell lysates by immunoprecipitation with an antibody recognizing the intracellular domain of Tie2. The phosphorylation status of the 55 kDa Tie2 fragment was then examined by anti-phosphotyrosine immunoblotting of immunoprecipitates (Fig. 5B). The 55 kDa Tie2 fragment present at 24 h of PMA treatment was found to be tyrosine phosphorylated, however Ang1 treatment did not affect phosphorylation status of the endodomain (Fig. 5B).

As shown in figure 3A, 24 h of VEGF treatment stimulates formation of Tie2 endodomain. It was of interest therefore to examine whether this endodomain was phosphorylated. To do this endothelial cells were treated with VEGF for 24 h before stimulation with Ang1 for 30 min, followed by cell lysis, immunoprecipitation of Tie2 and probing the recovered Tie2 endodomain for tyrosine phosphorylation (Fig. 5C). As with PMA, VEGF stimulated Tie2 endodomain formation at 24 h and this was tyrosine phosphorylated, though Ang1 treatment did not enhance its phosphorylation (Fig. 5C).

## Discussion

In this study we have examined the effect different physiological activators have on relative levels of Tie receptors and Ang1 signalling. Our data shows that cleavage of cellular Tie1 and Tie2 are both regulated by VEGF but with different kinetics. This results in an increase in cellular Tie2∶Tie1 ratio early after VEGF stimulation and a decrease in the ratio at later time points. In contrast, TNFα differentially affects the receptors by enhancing Tie1 loss whilst increasing Tie2 levels leading to a progressive increase in Tie2∶Tie1 ratio in cells treated with this ligand. Furthermore, this modulation of Tie1 and Tie2 by VEGF and TNFα affects Ang1 signalling, with a decrease in Tie1 relative to Tie2 enhancing Ang1-induced cellular Tie2 activation [17], [18], and decreased Tie2 suppressing cellular Tie2 activation [20].

The mechanisms responsible for the different time courses of Tie1 and Tie2 cleavage in response to PMA and VEGF are not known. However, it is possible that this reflects involvement of different proteases in cleavage of each of the receptors. Recently matrix metalloprotease-14 (MMP14) has been implicated in mediating Tie2 cleavage and the increase in PMA-activated Tie2 cleavage correlates with induction of increased MMP14 expression [21]. Thus the delay in PMA and VEGF-induced cleavage of Tie2 may be due to the requirement for induction of transcription and translation of the protease responsible. Although the protease mediating Tie1 cleavage has not yet been identified, the rapid activation of cleavage by PMA and VEGF suggests the protease responsible for Tie1 cleavage does not require induction of expression for it to act on the receptor.

The ability of TNFα to cause a time dependent increase in Tie2∶Tie1 ratio in endothelial cells was found to involve both effects on Tie1 cleavage and effects on expression of Tie1 and Tie2. As shown in figure 2, TNFα does stimulate cleavage of Tie1 at early time points. However at 24 h of TNFα-treatment the loss of Tie1 is not restricted to surface expressed full-length Tie1 but also seen for the precursor form and is not accompanied by the presence of Tie1 endodomain, indicating effects on Tie1 expression rather than cleavage at this time point. In contrast, TNFα failed to induce Tie2 cleavage but rather enhanced expression of Tie2. The ability of TNFα to stimulate expression of Tie2 has also been observed by others [27]. It will be of interest in future studies to examine the mechanisms responsible for TNFα-induced Tie2 expression and suppression of Tie1. Irrespective of the mechanism, the data clearly show TNFα causes a time dependent increase in cellular Tie2∶Tie1 ratio which is particularly marked at 24 h.

Together the findings of the present study show that the molecular balance between cellular Tie2∶Tie1 is dynamically regulated by pathophysiologically relevant factors and this balance influences the ability of Ang1 to signal in endothelial cells. These findings identify the Tie2∶Tie1 ratio as a key determinant of angiopoietin signalling in endothelial cells. Interestingly, Ang1 is considered to provide a fairly constant maintenance signal to endothelial cells, is present bound to extracellular matrix and is produced by perivascular cells at a relatively constant rate [9]. In the presence of this constitutively expressed Ang1 ligand negative regulation of Ang1 signalling can occur via the antagonist activity of Ang2 [3], [9], which is dynamically regulated by a range of factors including hypoxia, VEGF and angiotensin II [32], [33]. The present findings suggest an additional mechanism of controlling Ang1 signalling at the level of the Tie2∶Tie1 complex by modification of the cellular Tie2∶Tie1 ratio. The ability of physiological stimuli to regulate this ratio, in both directions, may be important for integrating angiopoietin signalling with other signals in the endothelial microenvironment.

The focus of the present study has been to examine the effects of VEGF and TNFα on the relative levels of Tie1 and Tie2 within the same population of endothelial cells and the effects on Ang1 signalling. Clearly it will be important to determine the functional significance of the effects of VEGF and TNFα on Ang1 signalling *in vivo*. A prediction from the cellular findings is that *in vivo* Ang1 signalling under basal conditions is limited by the responsiveness of the Tie2 receptor, which is determined by the Tie2∶Tie1 ratio in the cell, to the pool of perivascular Ang1. Furthermore, short-term increases in local VEGF concentration would enhanced Tie2 responsiveness and increase Ang1 signalling. This would allow the endothelium to maintain a quiescent state due to the presence of perivascular Ang1 without requiring maximal signalling through Tie2 under normal basal conditions whilst having the ability to enhance this pro-quiescent signal by increasing Tie2 responsiveness to Ang1 in the presence of transient increases in VEGF *in vivo*. Such a mechanism of local regulation of Tie2 responsiveness by VEGF would prevent short-term fluctuations in VEGF from causing vascular destabilization without having to maintain constant maximal levels of Ang1 signalling in the endothelium. The endothelium would retain the ability to respond acutely to VEGF if there was a coincident increase in Ang2. In addition, chronic VEGF elevation would also be expected to result in vessel destabilization as the enhancement of Tie2 responsiveness to Ang1 does not occur under conditions of long-term VEGF treatment. Whilst it is possible to speculate on the functional significance of the cellular effects *in vivo* it will be important in future work to test these hypotheses and examine the significance of the cellular changes in receptors and signalling in pathophysiological situations *in vivo* such as during angiogenesis.

In conclusion this study has shown the levels of intact Tie2 and Tie1 in endothelial cells are differentially controlled in both the short- and long-term by factors regulating ectodomain cleavage of the two receptors. This results in modulation of the molecular Tie2∶Tie1 balance in cells by various stimuli. Furthermore, the molecular balance between these two receptors determines the level of Ang1-induced Tie2 activation in the cell. These findings highlight the importance of regulation of signalling at the level of the receptor. Such control may be an important adaptation to allow modulation of cellular signalling responses in systems in which the activating ligand is normally present in excess or where the ligand provides a constitutive maintenance signal.

## Materials and Methods

### Cells

Human umbilical vein endothelial cells (HUVEC) were from Promocell and were maintained in Medium 199 containing 20% foetal calf serum, 5 units/ml heparin, and 50 µg/ml endothelial cell growth supplement. As indicated in Results, cells were treated with the following concentrations of reagents: PMA, 10 ng/ml (Merck, Nottingham, UK); VEGF, 100 ng/ml (PeproTech, London, UK); TNFα, 100 ng/ml (PeproTech, London, UK); Ang1 200 ng/ml (R&D Systems, Abingdon, UK), unless otherwise indicated. The γ-secretase inhibitor (IX, Merck, Nottingham,UK) was added prior to addition of the PMA and remained in the media throughout treatment. Annealed, purified and de-salted double-stranded siRNA oligonucleotides have been previously detailed [17] and were obtained from MWG Biotech (London, UK). For transfection with control scrambled siRNA, siRNA directed against Tie2 or Tie1, HUVEC were grown to approximately 80% confluence and then transfected with 100 nM siRNA using Lipofectamine as directed in the manufacturer's protocol 48 h before treatments and cell lysis. All other reagents were as described previously [34], [35].

### Immunoprecipitation and Immunoblotting

Cells were lysed with ice-cold lysis buffer (50 mM Tris, pH 7.4, 50 mM NaCl, 1 mM sodium fluoride, 1 mM EGTA, 1 mM sodium orthovanadate, 1% TritonX-100, complete protease inhibitor mixture) and lysates centrifuged at 13,000× g for 5 min to remove particulate matter.

For analysis of whole cell lysates, Laemmli sample buffer containing dithiothreitol, to produce a final concentration of 100 mM, was mixed with cleared lysates and boiled for 5 min before loading equal amounts of protein onto SDS-PAGE and resolving. For immunoprecipitates, supernatants cleared of particulate material by centrifugation at 13,000× g for 5 min were immunoprecipitated by the addition of 2 µg of the indicated antibody for2–3 h in the presence of protein-A beads. In some experiments Tie2 and Tie1 extracellular domain was immunoprecipitated from conditioned medium, in these cases medium was conditioned for 24 h and cleared by centrifugation before immunoprecipitation as for cell lysates. In all cases immunoprecipitated proteins were recovered by centrifugation at 13,000× g for 5 min and washed 3 times with wash buffer (as lysis buffer but with 0.1% Triton X-100). Proteins were eluted from beads by the addition of Laemmli sample buffer containing 100 mM dithiothreitol and boiling for 5 min before SDS-PAGE. For immunoblotting proteins were transferred to nitrocellulose membranes electrophoretically and membranes probed with Tie1, Tie2 or phospho-Tie2 antibodies as appropriate. Blots were stripped and re-probed with anti-tubulin. Immunoreactive proteins were visualized with peroxidase-conjugated secondary antibodies and chemiluminescent detection [36], quantification of blots was by densitometric scanning and scans from each track were normalized against tubulin.

### Data Analysis

Bands on Western blots were quantified by densitometric scanning of films. Graphs were derived from 3 or more independent experiments and data is plotted as means and standard error. Statistical analysis was performed using Student's ‘t’ test and differences between means judged statistically significant for p<0.05.
